# Evaluation of Drug Interactions in Hospitalized Patients with Respiratory Disorders in Greece

**DOI:** 10.3390/arm91010008

**Published:** 2023-02-05

**Authors:** Marios Spanakis, Petros Ioannou, Sotiris Tzalis, Flora Chouzouri, Evridiki Patelarou, Diamantis P. Kofteridis, Katerina M. Antoniou, Sophia E. Schiza, Athina Patelarou, Nikos Tzanakis

**Affiliations:** 1 Department of Nursing, School of Health Sciences, Hellenic Mediterranean University, 71004 Heraklion, Crete, Greece; 2 Computational Biomedicine Laboratory, Institute of Computer Science, Foundation for Research & Technology-Hellas (FORTH), GR-70013 Heraklion, Crete, Greece; 3 Department of Internal Medicine & Infectious Diseases, University Hospital of Heraklion, 71110 Heraklion, Crete, Greece; 4 Department of Respiratory Medicine, University Hospital of Heraklion, Medical School, University of Crete, 71003 Heraklion, Crete, Greece; 5 Sleep Disorders Unit, Department of Respiratory Medicine, Medical School, University of Crete, 71003 Heraklion, Crete, Greece

**Keywords:** drug–drug interactions, DDIs, chronic obstructive pulmonary disease, COPD, asthma, chronic respiratory diseases, pneumonia, adverse drug reactions, ADRs

## Abstract

**Highlights:**

**What are the main findings?**

- Patients admitted for hospitalization in Greece due to respiratory disorders are patients with multimorbidity, polypharmacy, and a high prevalence of drug–drug interactions (DDIs) in their medication regimens.
- Clinically significant DDIs that may require modulation in medical regimen or patient monitoring for side effects accounted for 58% upon admission and discharge and less during hospitalization (43%).

**What are the implications of the main findings?**

- The recorded DDIs mostly refer to cases requiring monitoring and caution to avoid the oc-currence of QT-prolongation, INR modulation, and CYP-mediated metabolism inhibition.
- The clinical significance of DDIs within the cohort can be considered manageable under proper patient monitoring, but clinicians should be aware and always examine if any oc-curring arrhythmias, INR modulations, and prolonged or increased drug actions are linked with DDIs.

**Abstract:**

*Background*: Patients with respiratory disorders often have additional diseases and are usually treated with more than one medication to manage their respiratory conditions as well as additional comorbidities. Thus, they are frequently exposed to polypharmacy (≥5 drugs), which raises the risk for drug–drug interactions (DDIs) and adverse drug reactions (ADRs). In this work, we present the results regarding the prevalence of DDIs in hospitalized patients with respiratory disorders in Greece. *Methods*: A 6-month descriptive single-center retrospective observational study enrolled 102 patients with acute or chronic respiratory disorders. Clinical characteristics and medication regimens were recorded upon admission, hospitalization, and discharge. The prevalence of DDIs and their clinical significance was recorded and analyzed. *Results*: Unspecified acute lower respiratory tract infection (25%), exacerbations of chronic obstructive pulmonary disease (12%) and pneumonia (8%) were the most frequent reasons for admission. Cardiovascular disorders (46%), co-existing respiratory disorders (32%), and diabetes (25%) were the most prevalent comorbidities. Polypharmacy was noted in 61% of patients upon admission, 98% during hospitalization, and 63% upon discharge. Associated DDIs were estimated to be 55% upon admission, 96% throughout hospitalization, and 63% on discharge. Pharmacodynamic (PD) DDIs were the most prevalent cases (81%) and referred mostly to potential risk for QT-prolongation (31.4% of PD-DDIs) or modulation of coagulation process as expressed through the international normalized ratio (INR) (29.0% of DDIs). Pharmacokinetic (PK) DDIs (19% of DDIs) were due to inhibition of Cytochrome P450 mediated metabolism that could lead to elevated systemic drug concentrations. Clinically significant DDIs characterized as “serious-use alternative” related to 7% of cases while 59% of DDIs referred to combinations that could be characterized as “use with caution—monitor”. Clinically significant DDIs mostly referred to medication regimens upon admission and discharge and were associated with outpatient prescriptions. *Conclusions:* Hospitalized patients with respiratory disorders often experience multimorbidity and polypharmacy that raise the risk of DDIs. Clinicians should be conscious especially if any occurring arrhythmias, INR modulations, and prolonged or increased drug action is associated with DDIs.

## 1. Introduction

Chronic respiratory diseases (CRDs) such as asthma, chronic obstructive pulmonary disease (COPD), emphysema, cystic fibrosis, bronchiectasis, etc. are a major burden to global health [1,2]. CRD patients also have a high incidence of comorbidities such as cardiovascular diseases (i.e., hypertension, coronary disease, etc.), diabetes, arthritis, and psychiatric conditions [3]. These conditions can further impair the quality of life for patients with CRDs and are often associated with higher mortality [4,5]. In addition, the recent pandemic crisis of coronavirus disease 2019 (COVID-19) introduced an additional risk factor since SARS-CoV-2, aside from causing the acute respiratory distress syndrome, can affect the respiratory system in a range of ways with long-term pulmonary complications that worsen or trigger diseases such as asthma, COPD, interstitial lung disease, lung fibrosis, etc. [6,7]. The management of CRDs requires an evidence-based guideline approach such as those of the global strategy for prevention, diagnosis, and management of COPD (GOLD guidelines) [8]. Generally, CRDs insufficiently controlled by monotherapy often require the incorporation of drug combinations with synergistic results to achieve optimum response [9]. Therefore, CRD patients are patients with multiple comorbidities and are often on polypharmacy to appropriately manage their respiratory disorder and the other comorbidities.

Polypharmacy is described as the concurrent administration of five or more medications. It is a widespread, complex situation among patients with chronic diseases and ia related with age, gender, and comorbidities as well as the use of over-the-counter (OTC) drugs and inappropriate health policies [10,11,12]. On the other hand, polypharmacy is also related to the increased risk of drug–drug interactions (DDIs) in cases of co-administration of drugs that share mutual or interconnected pharmacological pathways [13,14]. The modulation of absorption, metabolism, distribution, and elimination (ADME) processes may lead to pharmacokinetic DDIs (PK-DDIs) whereas modulation of primary or secondary pharmacological actions may result in pharmacodynamic DDIs (PD-DDIs). Consequently, this modulation of a drug’s pharmacological profile from a perpetrator drug can result in adverse drug reactions (ADRs) if a victim’s drug action is enhanced or treatment failure if it is reduced [15].

In previous years, there were several studies that mainly focused on COPD and examined the incidence of polypharmacy in outpatients [9,16,17]. In addition, the impact of the current pandemic also set several alerts regarding potential DDIs in patients with respiratory disorders [18,19]. Throughout these studies, it was shown that polypharmacy, age, and multimorbidity, especially in COPD patients, may affect treatment response, as well as patients’ adherence and, overall, their quality of life. On the other hand, limited data is available regarding inpatients’ exposure to clinically significant DDIs in respiratory wards. In addition, the current pandemic of COVID-19 introduced another clinical field of potential DDIs that require attention within the hospital clinics [19,20]. The aim of this study was to evaluate the occurrence of DDIs in hospitalized patients with respiratory diseases from the perspective of their clinical significance and underlying pharmacological mechanisms.

## 2. Materials and Methods

### 2.1. Study Design and Ethics Approval

A descriptive single-center retrospective observational study was conducted over a 6-month period (January–June 2022) in the Department of Respiratory Medicine in the University Hospital of Heraklion in Crete, Greece. The study complied with the rules of the Declaration of Helsinki of 1975, as revised in 2013, and was in accordance with the General Data Protection Regulation (GDPR). The study was approved by both the Hellenic Mediterranean University (51/04-03-21) and the University Hospital of Heraklion ethics committee (16105/13-10-2021). The study guidelines for reporting observational studies (Strengthening the Reporting of Observational Studies in Epidemiology—STROBE) are presented in Table 1.

Patients that were admitted to the respiratory department of the hospital with diagnoses that referred to respiratory disorders according to International Classification of Diseases (ICD-10) were informed of the study’s objectives and enrolled upon freely and willingly signing the informed consent form. Patients that did not provide consent or did not understand the terms of participation were excluded from the study. Multimorbidity was considered for patients with more than 2 additional diseases apart from their diagnosis on admission. Patients with lung cancer disease as a reason for attendance were excluded. Anonymized demographics, clinical and laboratory data, and medication regiments were extracted from the hospital’s medical record system. All data were collected and analyzed anonymously, and no interventions were made regarding healthcare provision during hospitalization.

### 2.2. DDI Analysis

Polypharmacy was classified as co-administration of five or more (≥5) medications. Different pharmacologically active compounds within the same medicinal product were considered as different compounds and counted separately (i.e., ipratropium with albuterol). Medications obtained from the hospital’s medical record system were classified according to the Anatomical Therapeutic Chemical (ATC) classification system (Appendix A) and were presented with its second level (anatomical group-therapeutic subgroup, i.e., ATC- X00). DDIs were identified using available online drug interaction checker tools (Medscape and Drugs.com).

DDIs were characterized as pharmacokinetic (PK) or pharmacodynamic (PD) based on their underlying pharmacological mechanism. As for their significance, they were categorized as “Serious-Use alternative”, “Use with caution-Monitor”, and “Moderate-Minor” considering the level of evidence in the literature such as experts’ opinion, in silico/in vitro/in vivo data/, clinical studies, summary of product characteristics (SmPC), and regulatory reports [20,21,22]. The ATC groups paired in identified DDIs are represented as Circos diagrams generated with Circos Table Viewer v0.63-9^©^ (http://circos.ca/ accessed on 20 December 2022) [23].

### 2.3. Statistical Analysis

Data are presented as numbers or percentages for continuous variables and ex-pressed as mean values ± standard deviation (±SD) whereas for discrete variables (i.e., number of drugs) they are presented as median number with ± interquartile range. Statistically significant differences (*p* < 0.05) were assessed with Mann–Whitney t-test with 95% confidence intervals (CI) using GraphPad Prism version 8.0.1 for Windows, GraphPad Software, San Diego, CA, USA, www.graphpad.com accessed on 20 December 2022.

## 3. Results

### 3.1. Patient Demogrpahics, Diagnosis and Comorbidities, and Clinical Status

**Figure 1:**
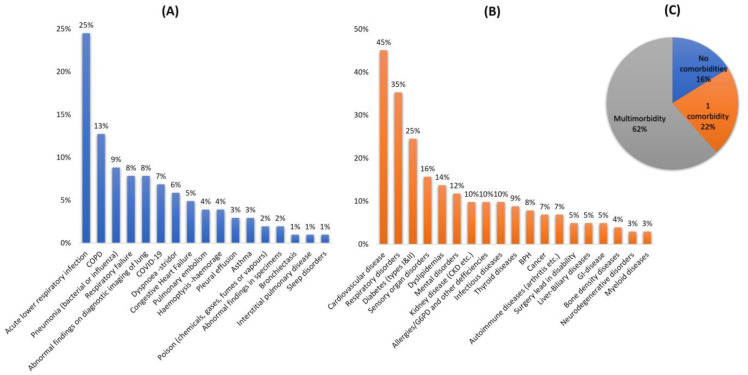
(**A**) ICD-10 diagnosis upon admission, (**B**) comorbidities recorded, and (**C**) patients’ burden of comorbidities within study’s cohort.

The study enrolled 102 patients (59% male, 43% female) admitted to the hospital’s respiratory ward with a diagnosed respiratory condition (Figure 1). Among them, 48% were residents in urban areas, the mean age was 69.2 years (±16.9), the mean BMI was 30.5 kg/m^2^, and 10% of them were habitually smokers (Table 2). The most common diagnoses upon admission were unspecified acute lower respiratory tract infection (25%), exacerbation of COPD (12%), and pneumonia (bacterial or influenza related) (8%). In addition, seven patients (7%) were hospitalized with respiratory complications after their recovery from COVID-19 (Figure 1A). Regarding comorbidities, cardiovascular diseases (46%), respiratory disorders (36%), diabetes (25%), sensory organs disorders (16%), dyslipidemias (14%), and mental disorders (12%) were the most frequent comorbidities (Figure 1B). Multimorbidity accounted for 38% within the cohort whereas 52% of admitted patients had one additional condition. Additional information for relevant clinical values of the participants can be found in the Supplementary Files.

### 3.2. Medications Administered

**Figure 2:**
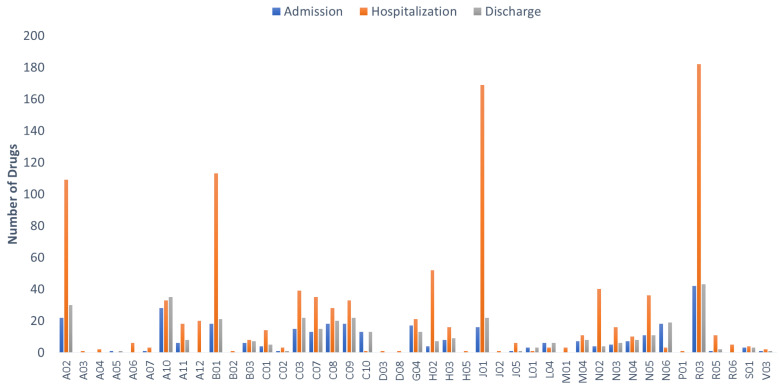
ATC drug categories that were administered in patients during admission (blue bars), hospitalization (red bars), and discharge (grey bars).

A median number of six drugs (IQR = 6) per patient were recorded upon admission, 11 (IQR = 5) during hospitalization, and seven (IQR = 7) upon discharge. Following the ATC categorization (2nd level), administered medications are organized into 43 different ATC groups (Figure 2). Upon admission, patients were mostly prescribed drugs for obstructive airway diseases (ATC-R03), antidiabetics (ATC-A10), and cardiovascular and lipid-modifying agent medications (ATC-C01, C03, C07, C08, C9, C10), as well as drugs for benign prostatic hyperplasia (ATC-G04). These categories, along with proton pump inhibitors (PPIs) (drugs for acid related disorders, ATC-A02), were also the most prevalent medications on discharge. During hospitalization, patients’ medication regimens were revised and modified according to treatment requirements. Hence, antibiotics (ATC-J01) usage increased along with administration of antithrombotic agents (ATC-B01), drugs for obstructive airway diseases (ATC-R03), and corticosteroids for systemic use (ATC-H02). For gastro-intestinal (GI) protection, PPIs were added, and there was a slight increase in usage of analgesics (ATC-N02) and anxiolytics (ATC-N05). The most administered drugs during hospitalization were esomeprazole (87%), enoxaparin (85%), ipratropium (60%), budesonide (46%), methylprednisolone (42%), salbutamol (40%), furosemide (32%), and insulin (27%). Regarding antibiotics during hospitalization, the β-lactam piperacillin (22%), the fluoroquinolone levofloxacin (20%), and the third generation cephalosporin ceftriaxone (18%) were the most administered. Overall, 141 different medications were recorded upon admission, 187 during hospitalization, and 146 on discharge.

### 3.3. Identified DDIs and Underlying Pharmacological Mechanisms

Polypharmacy accounted for 61% upon admission, 98% during hospitalization, and 63% on discharge (Figure 1A). The analysis of medication regimens revealed 453 cases from 252 different drug combinations identified as potential DDIs. Average values of two DDIs (min = 0, max = 7) upon admission, five DDIs (min = 0, max = 18) during hospitalization, and two DDIs (min = 0, max = 12) on discharge were identified. Table 3 and Table 4 present characteristic cases regarding PK-DDIs and PD-DDIs that were recorded. The full list of DDIs can be found in the Supplementary Files.

**Figure 3:**
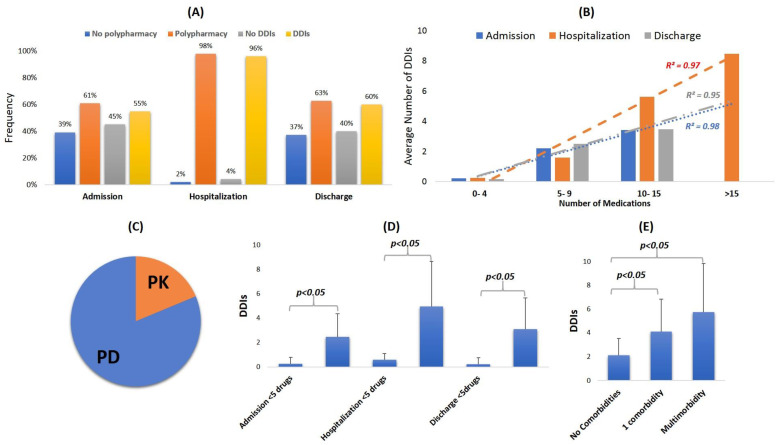
(**A**) Polypharmacy and DDI percentages recorded at each time point. (**B**) Correlation between number of medications and average number of DDIs identified. (**C**) Pharmacological mechanisms of DDIs. (**D**) DDIs as to polypharmacy at different time points. (**E**) DDIs as to disease burden. Statistically significant pairs are presented with brackets.

A higher number of DDIs was recorded during hospitalization (96%) compared to admission (55%) or discharge (60%) (Figure 1A). There was also a linear correlation (r^2^ > 0.95) in all time points between the numbers of medications administered as to the average number of DDIs identified (Figure 1B). The underlying pharmacological mechanisms of recorded DDIs were referring primarily to PD-DDIs (81%) (Figure 3C). Polypharmacy was related to higher occurrence of DDIs (*p* < 0.05, 95%) at all time points (Figure 3D). DDIs were also more prevalent (*p* < 0.05, 95% CI) in multimorbidity patients compared to those with no other underlying conditions (Figure 3E).

**Figure 4:**
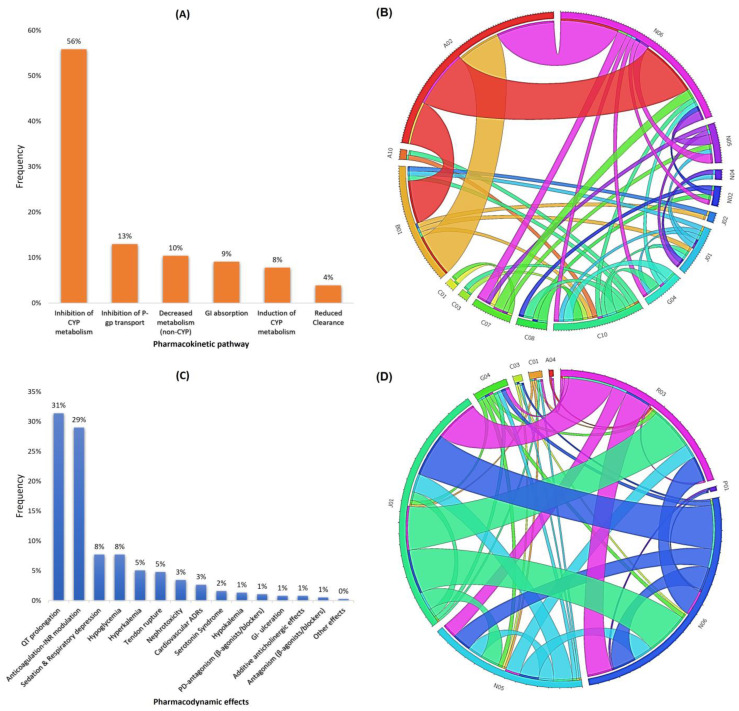
(**A**) Pharmacokinetic mechanisms of PK-DDIs; (**B**) Circos plot representing the ATC categories that can lead to CYP-mediated metabolism inhibition as recorded in this cohort; (**C**) Pharmacodynamic effects from the PD-DDIs; and (**D**) Circos plot representing ATC categories that can elevate the risk for QT-prolongation due to co-administration.

PK-DDIs refer to mechanisms involving inhibition of cytochrome P450 (CYP) mediated metabolism (56% of PK-DDIs), and, to a lesser extent, inhibition of P-glycoprotein mediated transport (13% of PK-DDIs) or other pathways (Figure 4A). Typical examples were the co-administration of CYP inhibitors such as chloramphenicol (ATC-J01), diltiazem (ATC-CO8), amlodipine (ATC-C08), itraconazole (ATC-J02), esomeprazole (ATC-A02), fluoxetine (ATC-N06), verapamil (ATC-C08), and amiodarone (ATC-C01) that potentially can elevate the concentrations of co-administered drug substrates for relative CYPs (i.e., CYP2C9, CYP2D6, CYP3A4). Similarly, co-administration of drugs that are P-gp inhibitors such as amiodarone, azithromycin, and carvedilol can inhibit the mediated transport of P-gp substrates (i.e., statins, dabigatran, etc.) changing their systemic concentration. The overall ATC associations that can lead to PK-DDIs due to inhibition of CYP-mediated metabolism (e.g., CYP3A4) are represented with a circos diagram (Figure 4B).

PD-DDIs referred mostly to drug combinations that increase the risk of QT-prolongation (31.4% of PD-DDIs); anticoagulation processes and INR-modulation (29.0%); synergism that enhances sedation and respiratory depression (7.7%); hypoglycemia (7.7%); and hyperkaliemia (5.1%) (Figure 4C). QT-prolongation risk often emerged from the co-administration of antibiotics (ATC-J01) (e.g., quinolones), drugs for obstructive airway diseases (ATC-R03) (e.g., selective β-2-adrenoreceptor agonists), antidepressants (ATC-N06) (e.g., selective serotonin reuptake inhibitors, SSRIs), and antipsychotics (ATC-N05) (e.g., diazepines and butyrophenone derivatives). The overall ATC associations that can lead to PD-DDIs of QT-prolongation are presented in a circos diagram (Figure 4D). A brief list of PK-DDIs and PD-DDIs is presented in Table 3 and Table 4, and the full PK-DDI and PD-DDIs table can be found in the Supplementary Files.

### 3.4. Clinical Significance of Identified DDIs

**Figure 5:**
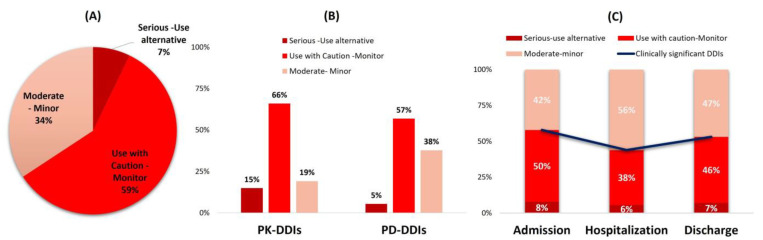
Distribution of recorded DDIs’ clinical significance (**A**) in all identified DDIs; (**B**) related to pharmacological mechanism involved; (**C**) in each time point.

Drug combinations with potentially clinically significant DDIs of “Serious-Use alternative” represented 7%, combinations of “Use with caution-Monitor” accounted for 59%, and of “Moderate-Minor Significance” represented 34% of all DDIs (Figure 5A). Considering significance and underlying pharmacological mechanisms, “Use with caution-Monitor” was related with 66% and 57% of PK- and PD-DDIs, respectively. “Serious-Use alternative” combinations were more common in PK-DDIs (15%) than PD-DDIs (5%), and the importance of “Moderate-Minor” DDIs were mostly for combinations resulting in PD-DDIs (38%) and less in PK-DDIs (19%) (Figure 5B). Considering the clinical significance related to the time point, “Serious-Use alternative” DDIs were recorded mostly upon admission (8%) and less during hospitalization (5%) or on discharge (7%) (Figure 5C). “Use-with caution-Monitor” DDIs were identified in 50% upon admission, 38% during hospitalization, and 46% on discharge. DDIs of “Moderate-Minor” significance were recorded in 42% of cases upon admission, 56% during hospitalization, and 47% on discharge. Overall, clinically significant DDIs (“Use alternative” and “Use with caution-Monitor”) accounted for 58% of the cases upon admission, 43% during hospitalization, and 52% on discharge (Figure 5C blue line).

## 4. Discussion

Polypharmacy and comorbidities, among other reasons, are contributing factors to the appearance of DDIs and ADRs. Generally, ADRs from DDIs decrease the quality of provided healthcare, prolong hospitalization as well as its costs, and deteriorate patients’ quality of life [24,25,26]. Hence, the awareness of potential DDIs among clinicians is an important factor that can assist healthcare teams to proceed to improved assessment and planning of healthcare provisions [27,28]. Considering respiratory diseases, the clinical information related to DDIs for hospitalized patients with respiratory disorders is lacking with most studies being oriented primarily toward COPD patients [9,29].

The current retrospective study presented the results regarding the prevalence of DDIs among 102 patients admitted to the respiratory department of the University Hospital of Heraklion in Greece. Compared to previous studies that focus on COPD or asthma, this work expanded the target group to include all potential cases of diagnosis according to the ICD-10 criteria for patients admitted to the respiratory department [30,31]. Moreover, the study recorded and analyzed the prevalence of DDIs in three discrete time points, upon admission, during hospitalization, and on discharge, hence, taking into consideration the prevalence of DDIs over time. The diagnoses upon admission in the current study were similar to those in the literature, suggesting acute or chronic lower respiratory disorders, infections, and pneumonia as the most common reasons for admission [32]. In addition, the cohort showed similar trends with previous studies examining the prevalence of comorbidities (66% multimorbidity within the cohort) with cardiovascular (46%) and respiratory disorders (36%) being the most prevalent co-existing conditions [3,16].

Regarding medication regimens, a high prevalence of polypharmacy was observed in hospital admission that intensified during hospitalization (Figure 1). The most administered medications during hospitalization were antibiotics, drugs for obstructive airway diseases, corticosteroids for systemic use, and PPIs for gastroprotection along with analgesics and anxiolytics. Polypharmacy has been described as a frequent situation and a risk factor for patients hospitalized for respiratory disorders such as acute exacerbation of COPD or respiratory tract infections (upper and lower) [31,33]. This also contributes to the excess number of drugs that these patients require during hospitalization (Figure 2). As a result, both multimorbidity and polypharmacy are associated with the exposure in drug combinations that potentially can lead to DDIs and consequently to ADRs [34]. At least one drug pair within each patients’ medication regimen that could result in DDIs was found in 55% upon admission, 96% throughout hospitalization, and 63% on discharge with a linear trend between the average number of DDIs and number of medications administered (Figure 3). In addition, comparable with previous studies for prescription practices and DDIs within the Greek healthcare ecosystem, results from the current work reveal a similar trend about polypharmacy and DDIs in outpatient prescriptions [17,20,21,22,35,36].

The pharmacological mechanisms of DDIs were due to synergistic actions on biological pathways (PD-DDIs) and to a lesser extent due to modulation of ADME processes (PK-DDIs). The majority of observed PD-DDIs involved elevation of QT-prolongation risk due to the co-administration of drugs that influence the repolarization phase of cardiac myocytes such as quinolones (ATC-J01), selective β-2-adrenoreceptor agonists (ATC-R03), and selective serotonin reuptake inhibitors (ATC-N06) (Table 4, Figure 4) [37,38,39,40]. The associated clinical risk for arrhythmias from co-administered drugs with QT-prolonging effects might be unpredictable: thus, when combining drugs with potentiating effect on QT interval, a risk–benefit analysis considering individual patient status along with each drug’s risk for QT-prolongation and monitoring for potential signs of arrhythmias is suggested [41,42,43]. The second most commonly observed PD-DDI was the potential modulation of anticoagulation from a combination of drugs that may increase INR, a PD-DDI of moderate significance that is easily managed and monitored within clinical routine [44,45,46]. The rest of the potential PD-DDIs were of lesser frequency, referring to combinations that may imbalance glucose or electrolyte levels, hence referring to cases easily addressed as part of patient monitoring during hospitalization [47,48]. The PD-DDIs considered to be serious referred to co-administration of linezolid with antidepressants, which raises the risk for serotonin syndrome, co-administration of drugs of the same ATC-category such as enoxaparin with dabigatran that can cause bleeding or abatacept with adalimumab (found on admission) that can lead to serious immunosuppression and respiratory tract infections in patients with history of lung disease [49,50,51,52]. In summary, PD-DDIs, although observed in higher frequency than PK-DDIs, were mostly manageable and within the risk–benefit analysis according to the medication protocols for this patient cohort. PK-DDIs were mostly related to inhibition of CYP-mediated metabolism such as CYP3A4, CYP2D6, CYP2C9, and CYP2C19. Typical examples of these PK-DDIs were the co-administration of PPIs, quinolones (e.g., ciprofloxacin), antidepressants (e.g., fluoxetine or paroxetine), and cardiovascular drugs such as Ca^2+^ channel blockers (e.g., verapamil or diltiazem) with CYP substrates [27,53]. PK-DDIs of drug combinations that should be avoided, thus requiring changes in medication regimens, referred mostly to CYP-mediated metabolism inhibition due to co-administration of CYP3A4 inhibitors (i.e., diltiazem, verapamil), CYP2C19 inhibitors (i.e., esomeprazole), P-gp inhibition (i.e., amiodarone), and CYP3A4 induction (i.e., primidone) [54,55,56]. These cases, although less frequently observed, could be associated with clinically significant DDIs due to the altered biotransformation of CYP substrates that could lead to modulation of their systemic concentrations outside their therapeutic window or reduce their action in case of administration as prodrugs (i.e., clopidogrel and PPIs).

Overall, the underlying pharmacological outcome of potential clinically significant DDIs of “Serious-Use alternative” made up 7% of total DDIs whereas 59% were of “Use with caution-Monitor” significance and 34% of “Moderate-Minor” importance. This is in line with previous observations regarding the prevalence of DDIs in CRD outpatients and considerations that despite the fact that these patients are usually under polypharmacy and multimorbidity conditions, the observed DDIs are mostly manageable and easily addressed if needed [57]. An additional factor that could contribute to the less frequent occurrence of clinically significant DDIs could be the low incidence of PK-DDIs for patients with respiratory disorders as in this cohort. The assessment of significance in cases of PK-DDIs is more feasible compared to PD-DDIs since they have specific and quantifiable mechanisms, whereas PD-DDIs have more complex mechanisms and clinical outcomes [58,59]. Nevertheless, considering that clinically significant DDIs are represented mostly with “Serious-Use alternative” and “Use with caution-Monitor”, an evident drop in clinically significant DDIs is observed during hospitalization compared to admission or discharge (Figure 5). This is expected for hospitalized patients under the healthcare provision of expert multidisciplinary medical teams that have additional clinical information (i.e., detailed laboratory values, medical imaging, etc.) and a full medication list at their disposal. Hence, they can proceed to better the risk–benefit analysis and therapy plan with fewer medication errors, avoid or better manage clinically significant DDIs, and, overall, provide better healthcare in accordance to evidence-based medical guidelines [60,61].

Some notable limitations regarding this study are the small sample size and that the study took place in one hospital. However, the clinical characteristics of the cohort correspond to data from the literature; hence, it can be argued that the pool of participants is representative for this patient cohort.

## 5. Conclusions

The present study explored the prevalence of DDIs for hospitalized patients with respiratory disorders in Greece. Unspecified acute lower respiratory tract infection was the main reason for admission. Patients often had an additional cardiovascular disorder along with their respiratory condition, so they were mostly under polypharmacy. A rise in administration of antibiotics, antithrombotic agents, drugs for obstructive airway diseases, and corticosteroids for systemic use was observed during hospitalization. As a result, multimorbidity and polypharmacy could be associated with potential DDIs. Pharmacological mechanisms of DDIs were due to synergistic PD effects that could lead to QT-prolongation or INR-modulation and to a lesser extent alteration of PK processes that could modulate drug concentrations such as inhibition of CYP-mediated metabolism. Despite the high frequency of polypharmacy and DDIs, their clinical significance was evaluated as manageable under proper patient monitoring whereas their frequency was lowered during hospitalization. In any case, clinicians in respiratory wards should be conscious and take into consideration that observed arrhythmias, INR modulations, and prolonged or increased drug action could be related to underlying DDIs in these patients.

## Figures and Tables

**Table 1:** STROBE information for the study regarding methods and results.

**Methods**	
Study design	Observational, retrospective, and descriptive study of DDIs
Setting	Patients hospitalized in the Respiratory Medicine Department
Participants	Patients requiring inpatient treatment for respiratory disorders
Variables	Demographic characteristics, clinical values, comorbidities, and medication regimens Analyze DDIs, their pharmacological mechanisms, and clinical significance
Data sources/ measurement	DDIs based on literature search and relative databases (Medscape, Drugs.com)
Study size	Target population: patients admitted with respiratory disordersStudy population: signed informed consent form
Bias	Diligence in informing the purpose and objectives of the study Diligence in recording the medication regimens in predefined time periods Recording demographics and medication regimens Analysis of data regarding the significance
**Results**	
Participants	102 patients that signed the informed consent
Descriptive data	Disease burden ○ No comorbidities: 16% ○ 1 comorbidity: 22% ○ Multimorbidity: 62% Average hospitalization days: 9 (median 7) Diagnosis upon admission: ○ Unspecified acute lower respiratory tract infection: 25% ○ COPD exacerbation: 12% ○ pneumonia (all types): 8% Number of medications per patient (median and IQR): ○ Admission 6 (6) ○ Hospitalization 11 (5) ○ Discharge 7 (7)
Outcome data	Comorbidities: ○ Cardiovascular disorders: 45% ○ Respiratory disorders: 35% ○ Diabetes: 25% Polypharmacy: ○ Admission: 61% ○ Hospitalization: 98% ○ Discharge: 63% 252 unique DDIs PK-DDIs: 18% and PD-DDIs: 82%
Main results	Multimorbidity patients: 62% Patients with at least one DDI: ○ Admission: 55% ○ Hospitalization: 98% ○ Discharge: 60% Linear correlation: number of medications vs number of DDIs Clinically significant DDIs: admission 58%; hospitalization 43%; discharge 53% Polypharmacy and multimorbidity associated with DDIs (*p* < 0.05, 95% CI) PD-DDIs: QT prolongation (31%), INR modulation (29%) PK-DDIs: inhibition of CYP-mediated metabolism (56%)

**Table 2:** Demographic characteristics of enrolled patients.

Demographics		Mean (±S.D)	Min/Max
Age (y)		69.3 (±16.9)	18/93
Height (m)		1.65 (±0.2)	1.5/1.9
Weight (kg)		77.4 (±18.2)	54.0/128.0
Body Mass Index (BMI, kg/m^2^)		30.5 (±5.4)	27.9/45.0
Comorbidities		2 (2) (median; IQR)	0/9
Hospitalization duration (d)		7 (5) (median, IQR)	2/74
**Residence**		**Social Habits**	
Urban	48%	Smoking	10%
Semi-urban	15%		
Rural	37		

**Table 3:** Drug pairs found in the study that can lead to pharmacokinetic drug interactions (Significance: SUA = Serious Use Alternative, Monitor: Use with Caution-Monitor; #: Frequency. Drug A: perpetrator, Drug B: victim or affected drug).

Drug A	ATC	Drug B (Victim)	ATC	#	Significance	Pharmacological Mechanism
Furosemide	C03	Allopurinol	M04	2	Monitor	Increased metabolite concentration
Amiodarone	C01	Digoxin	C01	1	SUA	P-gp inhibition
		Lovastatin		1	Monitor	
		Dabigatran	B01	2		
		Atorvastatin	C10	1		
		Carvedilol	C07	1		CYP2C9 inhibition
Carbamazepine	N03	Amitriptyline	N06	1	Monitor	Induction of CYP3A4
Itraconazole	J02	Apixaban	B01	1	Monitor	CYP3A4 inhibition
Carbamazepine	N03			1		Induction of CYP3A4
Azithromycin	J01	Lovastatin	C10	1	Monitor	CYP3A4 inhibition
		Dabigatran	B01	1		P-gp inhibition
Carvedilol	C07	Dabigatran	B01	1	Monitor	P-gp inhibition
Chloramphenicol	J01	Alfuzosin	G04	1	Monitor	CYP3A4 inhibition
		Haloperidol	N05	1		
		Rasagiline	N04	1		CYP1A2 inhibition
Esomeprazole	A02	Clopidogrel	B01	9	SUA	CYP2C19 inhibition
		Digoxin	C01	1	Monitor	P-gp inhibition
Diltiazem	C08	Eplerenone	C03	1	SUA	CYP3A4 inhibition
		Rivaroxaban	B01	1	Monitor	
		Pioglitazone	A10	1		
		Tamsulosin	G04	2		
		Donepezil	N06	1		
Omeprazole	A02	Escitalopram	N06	1	Monitor	CYP2C9 inhibition
Esomeprazole				7	Monitor	
Lovastatin	C10	Dabigatran	B01	1	Monitor	P-gp inhibition
Haloperidol	N05	Nebivolol	C07	1	Monitor	CYP2D6 inhibition
Paroxetine	N06	Aripiprazole	N05	1	Monitor	CYP2D6 inhibition
Primidone	N03	Roflumilast	R03	1	SUA	Induction of CYP3A4
Sertraline	N06	Metoprolol	C07	2	Monitor	CYP2D6 inhibition
Amlodipine	C08	Simvastatin	C10	1	SUA	CYP3A4 inhibition
Sulfomathoxazole	J01	Acenocoumarol	B01	1	Monitor	CYP2C9 inhibition
Fluoxetine	N06	Tamsulosin	G04	1	Monitor	CYP2D6 inhibition
Amlodipine	C08	Tramadol	N02	1	Monitor	CYP3A4 inhibition
Verapamil	C08	Simvastatin	C10	1	SUA	CYP3A4 inhibition
		Rivaroxaban	B01	1	Monitor	P-gp inhibition

**Table 4:** Drug pairs found in the study that can lead to pharmacodynamic drug interactions due to additive, synergistic, or antagonistic effects (Significance: SUA = Serious Use Alternative, Monitor: Use with Caution-Monitor; #: Frequency).

Drug A	ATC	Drug B	ATC	#	Significance	Pharmacological Mechanism
Abatacept	L04	Adalimumab	L04	1	SUA	Immunosuppression
Alfuzosin	G04	Haloperidol	N05	1	Monitor	QT prolongation
		Salbutamol	R03	2		
Allopurinol	M04	Acenocoumarol	B01	1	Monitor	INR modulation
Amiodarone	C01	Azithromycin	J01	1	Monitor	QT prolongation
		Quetiapine	N05	1		
Amitriptyline	N06	Indapamide	C03	1		
Aspirin	B01	Duloxetine	N06	1	Monitor	INR modulation
Azithromycin	J01	Olanzapine	N05	2	Monitor	QT prolongation
		Mirtazapine	N06	1		
		Formoterol	R03	1		
Bisoprolol	C07	Rivastigmine	N06	1	SUA	Cardiovascular ADRs
		Tramadol	Ν02	1		
Ciprofloxacin	J01	Haloperidol	N05	1	Monitor	QT prolongation
		Salbutamol	R03	4		
		Alfuzosin	G04	1		
Citalopram	N06	Levofloxacin	J01	1	Monitor	QT prolongation
Clonazepam	N03	Bromazepam	N05	1	Monitor	Sedation, respiratory depression
Donepezil	N06	Escitalopram	N06	2	Monitor	QT prolongation
		Levofloxacin	J01	1		
		Salbutamol	R03	2		
		Haloperidol	N05	1		
		Alfuzosin	G04	1		
		Nintedanib	L01	1		
		Ceftriaxone	J01	9		
		Methylprednisolone	H02	26		
		Apixaban	B01	1	Monitor	INR modulation
Escitalopram	N06	Haloperidol	N05	1	Monitor	QT prolongation
		Salbutamol	R03	2		
		Levofloxacin	J01	4		
Gabapentin	N03	Fentanyl	N02	1	SUA	Sedation, respiratory depression
		Lorazepam	N05	1		
Hydroxychloroquine	P01	Citalopram	N06	1	Monitor	QT prolongation
Hydroxyzine	N05	Solifenacin	G04	1	Monitor	
Leflunomide	L04	Acenocoumarol	B01	1	Monitor	INR modulation
Levofloxacin	J01	Olanzapine	N05	1	Monitor	QT prolongation
		Mirtazapine	N06	1		
		Trimethoprim	J01	2		
		Salbutamol	R03	5		
		Alfuzosin	G04	1		
Levomepromazine	N05	Trifluoperazine	N05	1	Monitor	QT prolongation
Linezolid	J01	Salbutamol	R03	3	Monitor	Cardiovascular ADRs
		Fentanyl	N02	1	SUA	Serotonin syndrome
		Quetiapine	N05	1		
		Citalopram	N06	1		
		Insulin	A12	1	Monitor	Hypoglycemia
Mirtazapine	N06	Olanzapine	N05	1	Monitor	QT prolongation
		Venlafaxine	N06	3		
		Rivaroxaban	B01	1	Monitor	INR modulation
		Moxifloxacin	J01	1	Monitor	QT prolongation
Perindopril	C09	Allopurinol	M04	1	SUA	Anaphylaxis risk, Steven Johnson’s syndrome
Perphenazine	N05	Levomepromazine	N05	1	Monitor	QT prolongation
		Indapamide	C03	1		
Piperacillin	J01	Vancomycin	J01	1	Monitor	Risk of nephrotoxicity
Potassium Chloride	A12	Eplerenone	C03	1	SUA	Hyperkalemia
Pregabalin	N03	Lorazepam	N05	1	Monitor	Sedation, respiratory depression
Quetiapine	N05	Donepezil	N06	1	Monitor	QT prolongation
		Levodopa	N04	1		
		Levofloxacin	J01	2		
Ramipril	C09	Potassium Chloride	A12	1	Monitor	Hyperkalemia
Ipratropium	R03	Risperidone	N05	1	Monitor	Hypoglycemia
Salbutamol		Haloperidol	N05	4	Monitor	QT prolongation
		Propafenone	C01	1		
		Azithromycin	J01	7		
		Ondansetron	A04	1		
		Fluoxetine	N06	2		
Sertraline	N06	Salbutamol	R03	3	Monitor	QT prolongation
Spironolactone	C03	Potassium Chloride	A12	2	Monitor	Hyperkalemia
Tocilizumab	L04	Remdesivir	J05	1	Monitor	Hepatotoxicity

## Data Availability

During the data collection and analysis, all procedures were followed to ensure confidentiality of participants in accordance with EU directives and the General Data Protection Regulation (GDPR). Data presented in the study are available to use upon reasonable request/permission from the corresponding author. The data are not publicly available due to privacy statements and ethical reasons that were included in the informed consent form signed by the participants.

## Supplementary Materials

The following supporting information can be downloaded at: https://www.mdpi.com/article/10.3390/arm91010008/s1, Figure S1: Gender, age distribution and clinical values for kidney and liver function as extracted from the medical records from participants in the study. CRP: C-reactive protein, SGPT: serum glutamic-pyruvic transaminase, SGOT: serum glutamic oxaloacetic transaminase, AL: Alkaline phosphatase; Table S1: Drug combinations that may lead to pharmacokinetic DDIs as they are recorded in the current study. SUA: Serious-Use alternative, Monitor: Use with caution-Monitor, Moderate: Moderate-Minor, frequency: number of cases. Table S2: Drug combinations that may lead to pharmacodynamic DDIs as they are recorded in the current study. SUA: Serious-Use alternative, Monitor: Use with caution-Monitor, Moderate: Moderate-Minor, frequency: number of cases.

## Appendix A

Drugs were classified and presented in figures and tables according to the Anatomical Therapeutic Chemical (ATC) classification system.

**Table A1:** Pharmacological subgroups of drugs that were classified into the relative ATC-groups.

ATC	Drug Category	Pharmacological Subgroup
A02	Drugs for acid-related disorders	Proton pump inhibitors
A03	Drugs for functional gastrointestinal disorders	Propulsives
A04	Antiemetics and antinauseants	Serotonin (5-HT3) antagonists
A05	Bile and liver therapy	Bile acids and derivatives
A06	Drugs for constipation	Softeners, emollients; Osmotically acting laxatives
A07	Antidiarrheal and intestinal anti-inflammatory/anti-infective agents	Antipropulsives; Antidiarrheal micro-organisms
A10	Drugs used in diabetes	Biguanides; Dipeptidyl peptidase 4 (DPP-4) inhibitors; Sodium-glucose co-transporter 2 (SGLT2) inhibitors
A11	Vitamins	Vitamin B-complex
A12	Mineral supplements	Calcium, combinations with vitamin D; Potassium; Magnesium
B01	Antithrombotic agents	Vitamin-K antagonists; Heparin group; Direct oral anticoagulants
B02	Antihemorrhagics	Vitamin K and other hemostatics
B03	Antianemic preparations	Ferrous supplements
C01	Cardiac therapy	Cardiac glycosides; antiarrhythmics; Vasodilators
C02	Antihypertensives	Imidazoline receptor agonists
C03	Diuretics	Thiazides; Sulfonamides; Aldosterone antagonists
C07	β-blockers	selective β-blockers; α- and β-blockers
C08	Ca^2+^ channel blockers	CCBs with vascular effects; CCBs with direct cardiac effects
C09	Agents acting on the renin–angiotensin system	ACE inhibitors; ARBs
C10	Lipid modifying agents	HMG CoA reductase inhibitors (statins)
D03	Preparations for treatment of wounds and ulcers	Cicatrizants
D08	Antiseptics and disinfectants	Antiseptics and disinfectants
G04	Urologicals	Drugs used in benign prostatic hypertrophy
H02	Corticosteroids for systemic use	Glucocorticoids
H03	Thyroid therapy	Thyroid hormones
H05	Calcium homeostasis	Antiparathyroid agents
J01	Antibacterials for systemic use	Penicillin; macrolides; quinolones
J02	Antimycotics for systemic use	Imidazole and Triazole derivatives
J05	Antivirals for systemic use	Direct acting antiviral drugs (remdesivir)
L01	Antineoplastic agents	Protein kinase inhibitors
L04	Immunosuppressant	Interleukin inhibitors; Antimetabolites
M01	Anti-inflammatory and antirheumatic products	Anti-inflammatory and antirheumatic products, non-steroids
M04	Antigout preparations	Uric acid inhibitors
N02	Analgesics	Analgesics and antipyretics
N03	Antiepileptic	Carboxamide (carbamazepine) and fatty acid (valproic acid) derivatives
N04	Anti-Parkinson drugs	Dopaminergic agents
N05	Psycholeptic	Antipsychotics; anxiolytics; sedatives
N06	Psychoanaleptics	Antidepressants
P01	Antiprotozoal	Aminoquinolines
R03	Drugs for obstructive airway diseases	β-2-receptor agonists; glucocorticoids
R05	Cough and cold preparations	Expectorants; Mucolytics
R06	Antihistamines for systemic use	Aminoalkyl ethers; Substituted alkylamines
S01	Ophthalmological	Antibiotics; β-blocking agents
V03	All other therapeutic products	Detoxifying agents for antineoplastic treatment
