# Supplementary material for: Evaluation of Drug Interactions in Hospitalized Patients with Respiratory Disorders in Greece
Source: Adv Respir Med. 2023 Feb 5;91(1):74–92. doi: 10.3390/arm91010008 (PMC9952796; doi:10.3390/arm91010008)
Supplement: Supplementary file 1 [file arm-91-00008-s001.zip › arm-2141880-supplementary.pdf]

Evaluation of Drug Interactions in Hospitalized Patients with Respiratory Disorders in Greece

Marios Spanakis [1,2,*], Petros Ioannou [3], Sotiris Tzalis [3], Flora Chouzouri [3], Evridiki Patelarou [1], Diamantis P. Kofteridis [3], Katerina M. Antoniou [4], Sophia E. Schiza [5], Athina Patelarou [1] and Nikos Tzanakis [4]

[1] Department of Nursing, School of Health Sciences, Hellenic Mediterranean University, 71004 Heraklion, Crete, Greece
[2] Computational Biomedicine Laboratory, Institute of Computer Science, Foundation for Research & Technology-Hellas (FORTH), GR-70013 Heraklion, Crete, Greece
[3] Department of Internal Medicine & Infectious Diseases, University Hospital of Heraklion, 71110 Heraklion, Crete, Greece
[4] Department of Respiratory Medicine, University Hospital of Heraklion, Medical, School, University of Crete, 71003 Heraklion, Crete, Greece
[5] Sleep Disorders Unit, Department of Respiratory Medicine, Medical School, University of Crete, 71003 Heraklion, Crete, Greece
* Correspondence: marspan@ics.forth.gr

The supplementary file contains additional information as supplementary tables (ST) regarding the scientific data that were presented in the manuscript

Figure S1 Gender, age distribution and clinical values for kidney and liver function as extracted from the medical records from participants in the study. CRP: C - reactive protein, SGPT: serum glutamic-pyruvic transaminase, SGOT: serum glutamic oxaloacetic transaminase, AL: Alkaline phosphatase

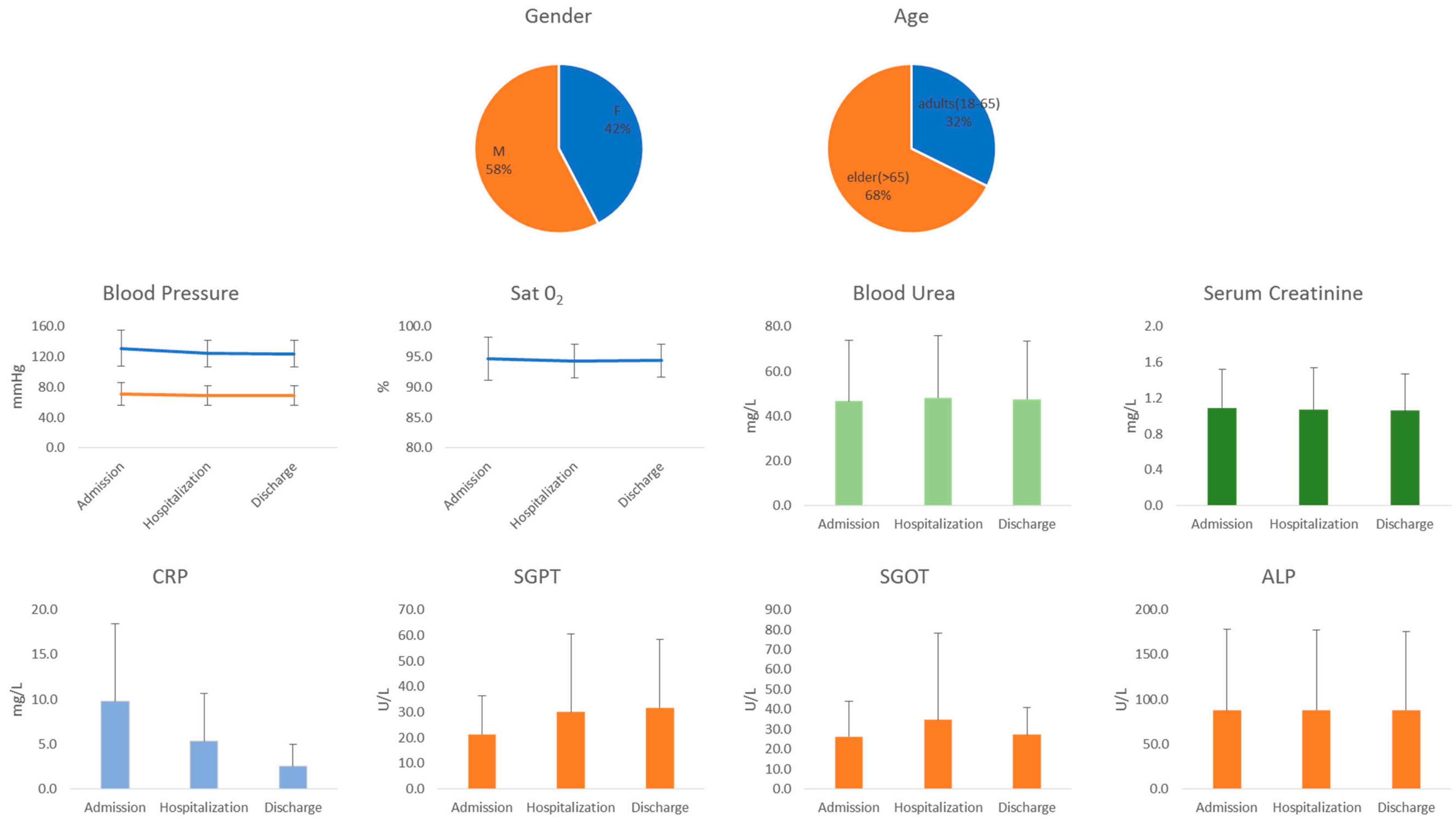

Table S1: Drug combinations that may lead in pharmacokinetic DDIs as they recorded in the current study. SUA: Serious-Use alternative, Monitor: use with caution-monitor, Moderate: moderate-minor, frequency: number of cases

Drug A	ATC	Drug B	ATC	Frequency	Significance	Description
Allopurinol	M04	Furosemide	C03	2	Monitor	Increased serum urate & Ct of oxypurinol
Amiodarone	C01	Atorvastatin	C10	1	Monitor	P-gp inhibition
Amiodarone	C01	Carvedilol	C07	1	Monitor	CYP2C9 inhibition
Amiodarone	C01	Dabigatran	B01	2	Monitor	P-gp inhibition
Amiodarone	C01	Digoxin	C01	1	SUA	P-gp inhibition
Amiodarone	C01	Lovastatin	C10	1	Monitor	P-gp inhibition
Amitriptyline	N06	Carbamazepine	N03	1	Monitor	Induction of CYP3A4 metabolism
Apixaban	B01	Carbamazepine	N03	1	Monitor	Induction of CYP3A4 metabolism
Apixaban	B01	Itraconazole	J02	1	Monitor	CYP3A4 inhibition
Azithromycin	J01	Dabigatran	B01	1	Monitor	P-gp inhibition
Azithromycin	J01	Lovastatin	C10	1	Monitor	CYP3A4 inhibition
Budesonide	R03	Silodosin	G04	1	Moderate	Induction of CYP3A4 metabolism
Carvedilol	C07	Dabigatran	B01	1	Monitor	P-gp inhibition
Chloramphenicol	J01	Alfuzosin	G04	1	Monitor	CYP3A4 inhibition
Chloramphenicol	J01	Haloperidol	N05	1	Monitor	CYP3A4 inhibition
Ciprofloxacin	J01	Metoprolol	C07	1	Moderate	Reduced Clearance
Ciprofloxacin	J01	Rasagiline	N04	1	Monitor	CYP1A2 ihnibition
Clopidogrel	B01	Esomeprazole	A02	9	SUA	CYP2C19 inhibition
Codeine	N02	Fluoxetine	N06	1	Moderate	CYP2D6 inhibition
Dexamethasone	H02	Dutasteride	G04	1	Moderate	Induction of CYP3A4 metabolism
Digoxin	C01	Esomeprazole	A02	1	Monitor	P-gp inhibition
Diltiazem	C08	Donepezil	N06	1	Monitor	CYP3A4 inhibition
Diltiazem	C08	Eplerenone	C03	1	SUA	CYP3A4 inhibition
Diltiazem	C08	Pioglitazone	A10	1	Monitor	CYP3A4 inhibition
Diltiazem	C08	Rivaroxaban	B01	1	Monitor	CYP3A4 inhibition
Diltiazem	C08	Tamsulosin	G04	2	Monitor	CYP3A4 inhibition
Enoxaparin	B01	Azithromycin	J01	8	Moderate	Decreased metabolism of enoxaparin
Escitalopram	N06	Omeprazole	A02	1	Monitor	CYP2C19 inhibition
Esomeprazole	A02	Citalopram	N06	3	Monitor	CYP2C9 inhibition

Esomeprazole	A02	Escitalopram	N06	7	Monitor	CYP2C9 inhibition
Ferrous gluconate	B03	Pantoprazole	A02	1	Moderate	GI absorption
Lovastatin	C10	Dabigatran	B01	1	Monitor	P-gp inhibition
Magnesium	A12	Ciprofloxacin	J01	1	Moderate	GI absorption
Magnesium	A12	Levofloxacin	J01	4	Moderate	GI absorption
Magnesium	A12	Levothyroxine	H03	1	Moderate	GI absorption
Nebivolol	C07	Haloperidol	N05	1	Monitor	CYP2D6 inhibition
Paroxetine	N06	Aripiprazole	N05	1	Monitor	CYP2D6 inhibition
Paroxetine	N06	Haloperidol	N05	1	Monitor	CYP2D6 inhibition
Primidone	N03	Fluticasone	R03	1	SUA	Induction of CYP3A4 metabolism
Primidone	N03	Roflumilast	R03	1	SUA	Induction of CYP3A4 metabolism
Sertraline	N06	Metoprolol	C07	2	Monitor	CYP2D6 inhibition
Simvastatin	C10	Amlodipine	C08	1	SUA	CYP3A4 inhibition
Sulfomathoxazole	J01	Acenocoumarol	B01	1	Monitor	CYP2C9 inhibition
Tamsulosin	G04	Fluoxetine	N06	1	Monitor	CYP2D6 inhibition
Tramadol	N02	Amlodipine	C08	1	Monitor	CYP3A4 inhibition
Verapamil	C08	Rivaroxaban	B01	1	Monitor	P-gp inhibition
Verapamil	C08	Simvastatin	C10	1	SUA	CYP3A4 inhibition

Table S2 Drug combinations that may lead in pharmacodynamic DDIs as they recorded in the current study. SUA: Serious-Use alternative, Monitor: use with caution-monitor, Moderate: moderate-minor, frequency: number of cases

Drug A	ATC	Drug B	ATC	Frequency	Significance	Description
Abatacept	L04	Adalimumab	L04	1	SUA	PD-synergism serious immunosuppression
Alfuzosin	G04	Formoterol	R03	1	Monitor	QT prolongation
Alfuzosin	G04	Haloperidol	N05	1	Monitor	QT prolongation
Alfuzosin	G04	Salbutamol	R03	2	Monitor	QT prolongation
Allopurinol	M04	Acenocoumarol	B01	1	Monitor	PD-synergism -anticoagulation -INR modulation
Alprazolam	N05	Quetiapine	N05	1	Moderate	PD-synergism, sedation & respiratory depression

Amiodarone	C01	Azithromycin	J01	1	Monitor	QT prolongation
Amiodarone	C01	Quetiapine	N05	1	Monitor	QT prolongation
Amitriptyline	N06	Indapamide	C03	1	Monitor	QT prolongation
Aripiprazole	N05	Haloperidol	N05	1	Monitor	QT prolongation
Aspirin	B01	Dexamethasone	H02	1	Moderate	GI- ulceration
Aspirin	B01	Duloxetine	N06	1	Monitor	PD-synergism -anticoagulation -INR modulation
Aspirin	B01	Enalapril	C09	1	Moderate	Risk of nephrotoxicity
Aspirin	B01	Furosemide	C03	1	Moderate	Risk of nephrotoxicity
Aspirin	B01	Lisinopril	C09	1	Moderate	Risk of nephrotoxicity
Aspirin	B01	Methylprednisolone	H02	1	Moderate	GI- ulceration
Aspirin	B01	Perindopril	C09	3	Moderate	Risk of nephrotoxicity
Aspirin	B01	Telmisartan	C09	1	Moderate	PD-synergism vasolidating effects
Atenolol	C07	Salbutamol	R03	1	Moderate	PD-antagonism (β-agonists/blockers)
Azithromycin	J01	Acenocoumarol	B01	1	Moderate	PD-synergism -anticoagulation -INR modulation
Azithromycin	J01	Formoterol	R03	1	Monitor	QT prolongation
Azithromycin	J01	Mirtazapine	N06	1	Monitor	QT prolongation
Azithromycin	J01	Olanzapine	N05	2	Monitor	QT prolongation
Azithromycin	J01	Quetiapine	N05	1	Monitor	QT prolongation
Azithromycin	J01	Venlafaxine	N06	1	Monitor	QT prolongation
Bisoprolol	C07	Rivastigmine	N06	1	SUA	PD-synergism cardiovascular side effects
Bromazepam	N05	Codeine	N02	4	Monitor	PD-synergism, sedation & respiratory depression
Bromazepam	N05	Tramadol	N02	1	Monitor	PD-synergism, sedation & respiratory depression
Carvedilol	C07	Salbutamol	R03	1	Moderate	PD-antagonism (β-agonists/blockers)
Cefixime	J01	Furosemide	C03	1	Moderate	Risk of nephrotoxicity
Ceftazidime	J01	Furosemide	C03	1	Moderate	Risk of nephrotoxicity

Ceftriaxone	J01	Furosemide	C03	3	Moderate	Risk of nephrotoxicity
Ciprofloxacin	J01	Alfuzosin	G04	1	Monitor	QT prolongation
Ciprofloxacin	J01	Haloperidol	N05	1	Monitor	QT prolongation
Ciprofloxacin	J01	Salbutamol	R03	4	Monitor	QT prolongation
Citalopram	N06	Acenocoumarol	B01	1	Moderate	PD-synergism -anticoagulation -INR modulation
Citalopram	N06	Dabigatran	B01	1	Monitor	PD-synergism -anticoagulation -INR modulation
Citalopram	N06	Furosemide	C03	1	Moderate	PD-synergism -anticoagulation -INR modulation
Citalopram	N06	Levofloxacin	J01	1	Monitor	QT prolongation
Clonazepam	N03	Bromazepam	N05	1	Monitor	PD-synergism, sedation & respiratory depression
Clopidogrel	B01	Escitalopram	N06	1	Moderate	PD-synergism -anticoagulation -INR modulation
Dexamethasone	H02	Amlodipine	C08	1	Moderate	Induction Na+ and fluid retention
Dexamethasone	H02	Ciprofloxacin	J01	1	Moderate	Risk of tendon rupture
Donepezil	N06	Alfuzosin	G04	1	Monitor	QT prolongation
Donepezil	N06	Escitalopram	N06	2	Monitor	QT prolongation
Donepezil	N06	Haloperidol	N05	1	Monitor	QT prolongation
Donepezil	N06	Levofloxacin	J01	1	Monitor	QT prolongation
Donepezil	N06	Moxifloxacin	J01	1	Monitor	QT prolongation
Donepezil	N06	Salbutamol	R03	2	Monitor	QT prolongation
Empagliflozin	A10	Furosemide	C03	1	Moderate	PD-synergism diuretic effects
Enoxaparin	B01	Apixaban	B01	1	Moderate	PD-synergism -anticoagulation -INR modulation
Enoxaparin	B01	Budesonide	R03	28	Moderate	PD-synergism -anticoagulation -INR modulation
Enoxaparin	B01	Candesartan	C09	1	Moderate	Hyperkalemia

Enoxaparin	B01	Ceftaroline	J01	5	Moderate	PD-synergism -anticoagulation -INR modulation
Enoxaparin	B01	Ceftriaxone	J01	9	Moderate	PD-synergism -anticoagulation -INR modulation
Enoxaparin	B01	Citalopram	N06	2	Moderate	PD-synergism -anticoagulation -INR modulation
Enoxaparin	B01	Clopidogrel	B01	1	Moderate	GI- ulceration
Enoxaparin	B01	Dabigatran	B01	1	SUA	PD-synergism -anticoagulation -INR modulation
Enoxaparin	B01	Dexamethasone	H02	2	Moderate	PD-synergism -anticoagulation -INR modulation
Enoxaparin	B01	Enalapril	C09	1	Moderate	Hyperkalemia
Enoxaparin	B01	Irbesartan	C09	1	Moderate	Hyperkalemia
Enoxaparin	B01	Lisinopril	C09	1	Moderate	Hyperkalemia
Enoxaparin	B01	Losartan	C09	1	Moderate	Hyperkalemia
Enoxaparin	B01	Methylprednisolone	H02	26	Moderate	PD-synergism -anticoagulation -INR modulation
Enoxaparin	B01	Nintedanib	L01	1	Moderate	PD-synergism -anticoagulation -INR modulation
Enoxaparin	B01	Olmesartan	C09	4	Moderate	Hyperkalemia
Enoxaparin	B01	Perindopril	C09	3	Moderate	Hyperkalemia
Enoxaparin	B01	Piperacillin	J01	13	Moderate	PD-synergism -anticoagulation -INR modulation
Enoxaparin	B01	Prednisolone	H02	2	Moderate	PD-synergism -anticoagulation -INR modulation
Enoxaparin	B01	Ramipril	C09	2	Moderate	Hyperkalemia
Enoxaparin	B01	Sulfamethoxazole	J01	1	Moderate	PD-synergism -anticoagulation -INR modulation
Enoxaparin	B01	Telmisartan	C09	2	Moderate	Hyperkalemia
Enoxaparin	B01	Valsartan	C09	3	Moderate	Hyperkalemia

Escitalopram	N06	Acenocoumarol	B01	1	Moderate	PD-synergism -anticoagulation -INR modulation
Escitalopram	N06	Ciprofloxacin	J01	1	Monitor	QT prolongation
Escitalopram	N06	Haloperidol	N05	1	Monitor	QT prolongation
Escitalopram	N06	Levofloxacin	J01	4	Monitor	QT prolongation
Escitalopram	N06	Moxifloxacin	J01	1	Monitor	QT prolongation
Escitalopram	N06	Salbutamol	R03	2	Monitor	QT prolongation
Fluoxetine	N06	Moxifloxacin	J01	1	Monitor	QT prolongation
Gabapentin	N03	Codeine	N02	1	Monitor	PD-synergism, sedation & respiratory depression
Gabapentin	N03	Escitalopram	N06	1	Moderate	PD-synergism, sedation & respiratory depression
Gabapentin	N03	Fentanyl	N02	1	SUA	PD-synergism, sedation & respiratory depression
Gabapentin	N03	Lorazepam	N05	1	Monitor	PD-synergism, sedation & respiratory depression
Hydroxychloroquine	P01	Citalopram	N06	1	Monitor	QT prolongation
Hydroxyzine	N05	Solifenacin	G04	1	Monitor	QT prolongation
Ibuprofen	M01	Furosemide	C03	1	Moderate	Risk of nephrotoxicity
Insulin	A10	Perindopril	C09	2	Moderate	PD-synergism hypoglycemia
Insulin	A10	Candesartan	C09	1	Moderate	PD-synergism hypoglycemia
Insulin	A10	Ciprofloxacin	J01	2	Moderate	PD-synergism hypoglycemia
Insulin	A10	Irbesartan	C09	1	Moderate	PD-synergism hypoglycemia
Insulin	A10	Moxifloxacin	J01	2	Moderate	PD-synergism hypoglycemia
Ipratropium	R03	Haloperidol	N05	3	Moderate	Additive anticholinergic effects,
Ipratropium	R03	Levomepromazine	N05	1	Moderate	PD-synergism hypoglycemia
Ipratropium	R03	Perphenazine	N05	1	Moderate	PD-synergism hypoglycemia
Ipratropium	R03	Trifluoperazine	N05	1	Moderate	PD-synergism hypoglycemia
Leflunomide	L04	Acenocoumarol	B01	1	Monitor	PD-synergism -anticoagulation -INR modulation

Levodopa	N04	Amlodipine	C08	1	Moderate	PD-synergism cardiovascular side effects
Levodopa	N04	Betaxolol	C07	1	Moderate	PD-synergism cardiovascular side effects
Levodopa	N04	Olmesartan	C09	1	Moderate	PD-synergism cardiovascular side effects
Levofloxacin	J01	Acenocoumarol	B01	1	Moderate	PD-synergism -anticoagulation -INR modulation
Levofloxacin	J01	Alfuzosin	G04	1	Monitor	QT prolongation
Levofloxacin	J01	Haloperidol	N05	1	Monitor	QT prolongation
Levofloxacin	J01	Insulin	A10	6	Moderate	PD-synergism hypoglycemia
Levofloxacin	J01	Mirtazapine	N06	1	Monitor	QT prolongation
Levofloxacin	J01	Olanzapine	N05	1	Monitor	QT prolongation
Levofloxacin	J01	Salbutamol	R03	5	Monitor	QT prolongation
Levofloxacin	J01	Sulfamethoxazole	J01	2	Monitor	QT prolongation
Levofloxacin	J01	Trimethoprim	J01	2	Monitor	QT prolongation
Levofloxacin	J01	Vildagliptin	A10	1	Moderate	PD-synergism hypoglycemia
Levomepromazine	N05	Trifluoperazine	N05	1	Monitor	QT prolongation
Linezolid	J01	Caffeine	N06	1	SUA	PD-synergism cardiovascular side effects
Linezolid	J01	Citalopram	N06	1	SUA	Serotonin syndrome
Linezolid	J01	Codeine	N02	2	SUA	PD-synergism, sedation & respiratory depression
Linezolid	J01	Escitalopram	N06	1	SUA	Serotonin syndrome
Linezolid	J01	Fentanyl	N02	1	SUA	Serotonin syndrome
Linezolid	J01	Fluoxetine	N06	1	Monitor	Serotonin syndrome
Linezolid	J01	Insulin	A12	1	Monitor	PD-synergism hypoglycemia
Linezolid	J01	Quetiapine	N05	1	SUA	Serotonin syndrome
Linezolid	J01	Salbutamol	R03	3	Monitor	PD-synergism cardiovascular side effects
Linezolid	J01	Tramadol	N02	1	SUA	Serotonin syndrome
Methylprednisolone	H02	Acenocoumarol	B01	2	Moderate	PD-synergism -anticoagulation -INR modulation
Methylprednisolone	H02	Ciprofloxacin	J01	6	Moderate	Risk of tendon rupture

Methylprednisolone	H02	Levofloxacin	J01	7	Moderate	Risk of tendon rupture
Methylprednisolone	H02	Moxifloxacin	J01	3	Moderate	Risk of tendon rupture
Mirtazapine	N06	Clonazepam	N03	1	Monitor	PD-synergism, sedation & respiratory depression
Mirtazapine	N06	Olanzapine	N05	1	Monitor	QT prolongation
Mirtazapine	N06	Paroxetine	N06	2	Monitor	QT prolongation
Mirtazapine	N06	Venlafaxine	N06	3	Monitor	QT prolongation
Moxonidine	C02	Bromazepam	N05	1	Moderate	PD-synergism, sedation & respiratory depression
Moxonidine	C02	Mirtazapine	N06	1	Moderate	PD-synergism, sedation & respiratory depression
Olanzapine	N05	Venlafaxine	N06	1	Monitor	QT prolongation
Olodaterol	R03	Furosemide	C03	1	Moderate	Hypokalemia
Paroxetine	N06	Acenocoumarol	B01	1	Moderate	PD-synergism -anticoagulation -INR modulation
Paroxetine	N06	Levofloxacin	J01	1	Monitor	QT prolongation
Paroxetine	N06	Moxifloxacin	J01	1	Monitor	QT prolongation
Paroxetine	N06	Quetiapine	N05	1	Monitor	PD-synergism, sedation & respiratory depression
Paroxetine	N06	Rivaroxaban	B01	1	Monitor	PD-synergism -anticoagulation -INR modulation
Paroxetine	N06	Sulfamethoxazole	J01	1	Monitor	QT prolongation
Paroxetine	N06	Trimethoprim	J01	1	Monitor	QT prolongation
Paroxetine	N06	Venlafaxine	N06	3	Monitor	QT prolongation
Perindopril	C09	Allopurinol	M04	1	SUA	Anaphylaxis risk, Steven Johnson's syndrome
Perindopril	C09	Metformin	A10	1	Moderate	PD-synergism hypoglycemia
Perphenazine	N05	Levomepromazine	N05	1	Monitor	QT prolongation
Perphenazine	N05	Trifluoperazine	N05	1	Monitor	QT prolongation
Perphenazine	N05	Indapamide	C03	1	Monitor	QT prolongation

Piperacillin	J01	Acenocoumarol	B01	1	Moderate	PD-synergism -anticoagulation -INR modulation
Piperacillin	J01	Vancomycin	J01	1	Monitor	Risk of nephrotoxicity
Potassium Chloride	A12	Eplerenone	C03	1	SUA	Hyperkalemia
Prednisolone	H02	Acenocoumarol	B01	1	Moderate	PD-synergism -anticoagulation -INR modulation
Prednisolone	H02	Ciprofloxacin	J01	1	Moderate	Risk of tendon rupture
Pregabalin	N03	Escitalopram	N06	1	Monitor	PD-synergism, sedation & respiratory depression
Pregabalin	N03	Fentanyl	N02	1	Monitor	PD-synergism, sedation & respiratory depression
Pregabalin	N03	Levocetirizine	R06	2	Monitor	PD-synergism, sedation & respiratory depression
Pregabalin	N03	Levomepromazine	N05	1	Monitor	PD-synergism, sedation & respiratory depression
Pregabalin	N03	Lorazepam	N05	1	Monitor	PD-synergism, sedation & respiratory depression
Primidone	N03	Bromazepam	N05	1	Monitor	PD-synergism, sedation & respiratory depression
Primidone	N03	Codeine	N02	1	Monitor	PD-synergism, sedation & respiratory depression
Quetiapine	N05	Aripiprazole	N05	1	Monitor	QT prolongation
Quetiapine	N05	Ciprofloxacin	J01	1	Monitor	QT prolongation
Quetiapine	N05	Donepezil	N06	1	Monitor	QT prolongation
Quetiapine	N05	Escitalopram	N06	1	Monitor	QT prolongation
Quetiapine	N05	Hydroxyzine	N05	1	Monitor	QT prolongation
Quetiapine	N05	Ipratropium	R03	8	Monitor	PD-synergism hypoglycemia
Quetiapine	N05	Levodopa	N04	1	Monitor	PD-antagonism (antidopaminergic activity)
Quetiapine	N05	Levofloxacin	J01	2	Monitor	QT prolongation

Quetiapine	N05	Melatonin	N05	1	Moderate	PD-synergism, sedation & respiratory depression
Quetiapine	N05	Mirtazapine	N06	1	Monitor	QT prolongation
Quetiapine	N05	Olanzapine	N05	1	Monitor	QT prolongation
Ramipril	C09	Potassium Chloride	A12	1	Monitor	Hyperkalemia
Risperidone	N05	Ipratropium	R03	1	Monitor	PD-synergism hypoglycemia
Salbutamol	R03	Azithromycin	J01	7	Monitor	QT prolongation
Salbutamol	R03	Ephedrine	C01	1	Monitor	PD-synergism cardiovascular side effects
Salbutamol	R03	Fluoxetine	N06	2	Monitor	QT prolongation
Salbutamol	R03	Formoterol	R03	1	Monitor	QT prolongation
Salbutamol	R03	Furosemide	C03	3	Moderate	Hypokalemia
Salbutamol	R03	Haloperidol	N05	4	Monitor	QT prolongation
Salbutamol	R03	Mirtazapine	N06	2	Monitor	QT prolongation
Salbutamol	R03	Moxifloxacin	J01	4	Monitor	QT prolongation
Salbutamol	R03	Olanzapine	N05	1	Monitor	QT prolongation
Salbutamol	R03	Ondansetron	A04	1	Monitor	QT prolongation
Salbutamol	R03	Propafenone	C01	1	Monitor	QT prolongation
Salbutamol	R03	Quetiapine	N05	1	Monitor	QT prolongation
Salbutamol	R03	Quetiapine	N05	1	Monitor	QT prolongation
Salmeterol	R03	Indapamide	C03	1	Monitor	Hypokalemia
Sertraline	N06	Acenocoumarol	B01	1	Moderate	PD-synergism -anticoagulation -INR modulation
Sertraline	N06	Alfuzosin	G04	1	Monitor	QT prolongation
Sertraline	N06	Salbutamol	R03	3	Monitor	QT prolongation
Sitagliptin	A10	Indapamide	C03	1	Moderate	PD-antagonism hyperglycemia
Spironolactone	C03	Potassium Chloride	A12	2	Monitor	Hyperkalemia
Tocilizumab	L04	Remdesivir	J05	1	Monitor	Induced hepatotoxicity
Topiramate	N03	Metformin	A10	1	Monitor	PD hyperchloremic metabolic acidosis

Tramadol	N02	Codeine	N02	1	SUA	PD-synergism, sedation & respiratory depression
Trazodone	N06	Escitalopram	N06	1	Monitor	QT prolongation
Trazodone	N06	Haloperidol	N05	1	Monitor	QT prolongation
Trazodone	N06	Levofloxacin	J01	1	Monitor	QT prolongation
Trazodone	N06	Salbutamol	R03	1	Monitor	QT prolongation
Venlafaxine	N06	Acenocoumarol	B01	1	Moderate	PD-synergism -anticoagulation -INR modulation
Venlafaxine	N06	Aripiprazole	N05	1	Monitor	PD-synergism, sedation & respiratory depression
Venlafaxine	N06	Haloperidol	N05	1	Monitor	QT prolongation
Venlafaxine	N06	Moxifloxacin	J01	1	Monitor	QT prolongation
Venlafaxine	N06	Quetiapine	N05	1	Monitor	PD-synergism, sedation & respiratory depression
Venlafaxine	N06	Sulfamethoxazole	J01	1	Monitor	QT prolongation
Venlafaxine	N06	Trimethoprim	J01	1	Monitor	QT prolongation
